# Open fracture of the acromion associated with a supraspinatus tendon rupture: an exceptional case report

**DOI:** 10.11604/pamj.2014.19.325.4793

**Published:** 2014-11-26

**Authors:** Abdelhak Mardy, Atif Mechchat, Amine El Ghazi, Mohammed El Idrissi, Mohammed Shimi, Abdelhalim El Ibrahimi, Abdelmajid El Mrini

**Affiliations:** 1 Department of Orthopedic Surgery B4, University Hospital Hassan II, Fez, Morocco

**Keywords:** Acromion, supraspinatus, open fracture

## Abstract

The combination of the acromion Open fracture to a section of the supraspinatus tendon is an exceptional situation. The author reports the case of a young patient with a wound of the posterolateral side of the right shoulder. Screwing was done for the fracture of the acromion after supraspinatus tendon suture with good clinical and radiological outcome after an appropriate rehabilitation.

## Introduction

Open fracture of the acromion is an exceptional clinical entity and should be reported. A heavy stab wound was the cause of this exceptional lesion association in a young patient whose surgical exploration noted a supraspinatus tendon rupture with capsular break. The reconstruction of the acromial arch with rotator cuff repair was needed urgently under antibiotic cover and anti tetanus serum.

## Patient and observation

**Figure 1:**
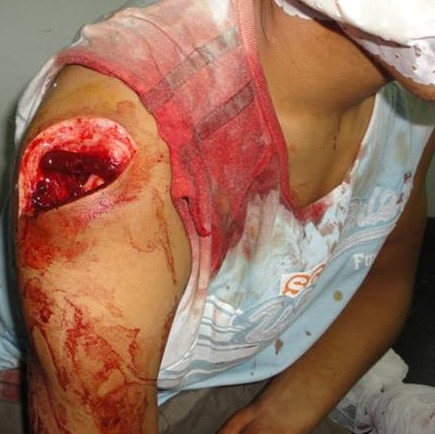
Clinical examination

The authors report the case of a 19 year old man; autonomous; right handed laterality; carpenter by profession; without medical or surgical particular history; victim of an accidental injury by a knife causing a wound from the outer side of the right shoulder. The clinical examination (Figure 1) objectified 5 cm wound on the posterolateral face of the shoulder associated with heavy bleeding and total functional impotence of the upper limb. Parenthesis has been noted on the outside and the vascular examination was normal. The shoulder joint was mobile and stable.

**Figure 2:**
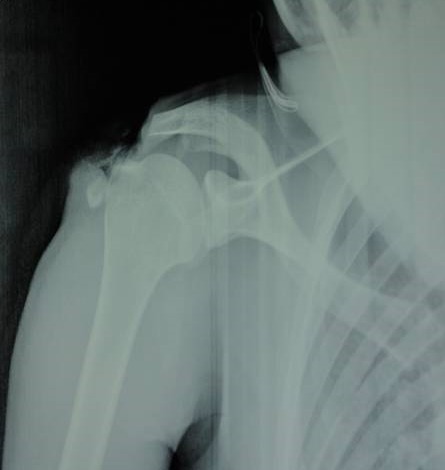
Radiological examination

**Figure 3:**
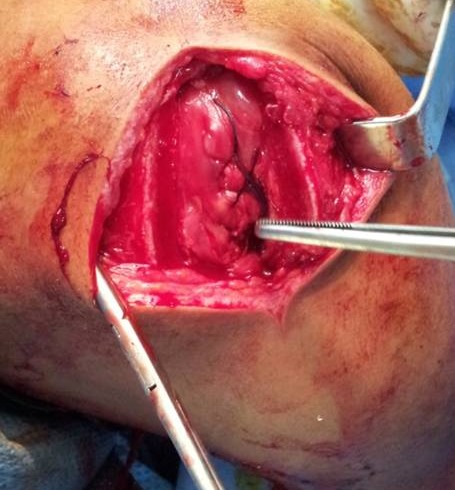
Surgical exploration

Radiographs (Figure 2) objectified a fracture of the acromion tupe III according to the classification of Kuhn and al. The appearance of the upper end of the humerus and the glenohumeral and acromioclavicular joint is without defects. Surgical exploration under general anesthesia found supraspinatus tendon break with an opening of the glenohumeral joint (Figure 3).

**Figure 4:**
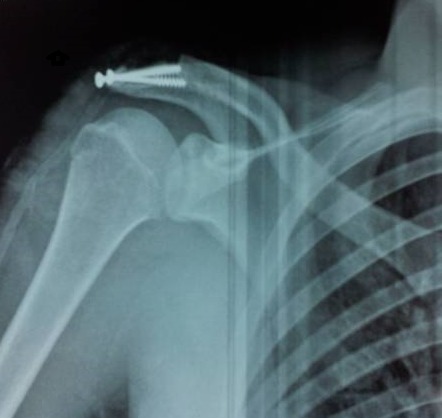
Radiological control

**Figure 5:**
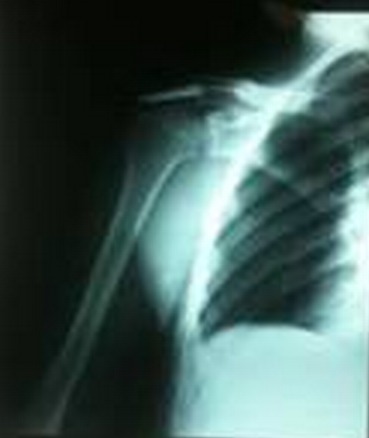
3 months later

A joint lavage was the first operative time. A careful closure of the joint capsule with absorbable suture followed by a suture supraspinatus tendon with a no absorbable suture by points X. An osteosynthesis of the acromion bone fragment by two 3.5 mm cancellous screws (Figure 4, Figure 5).

**Figure 6:**
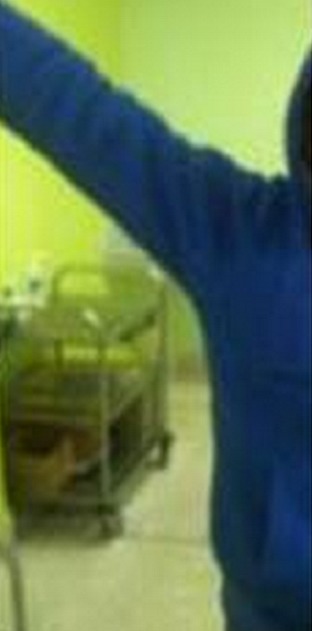
Clinical result

The evolution marked by a consolidation of the fracture with complete functional recovery after a suitable rehabilitation shoulder started working early and the joint amplitudes and muscle strength (Figure 6).

## Discussion

The literature review has not revealed a similar published case involving an open fracture of the acromion and supraspinatus tendon injury. The young person is more prone to this type of severe trauma to the shoulder and especially male [1, 2]. The management of this type of patient must respect the best practice treatment of open fractures of the skeleton [3] with washing and large spectrum antibiotic coverage with a cephalosporin second or third generation with an amino glycoside and metronidazole [4]. The injections of antitetanus serum and recall vaccination is also requiered. The rules of fixation of open fractures is also respected with a screw plug or a simple guy of this type of fracture of the acromion [5] and [6]. Tendon repair supraspinatus can be done by sutures in U or X types using a no absorbable synthetic yarn. The immobilization of the shoulder should not exceed 2O days [6]. The rehabilitation program begins by pendular movements of the shoulder followed by a working range of motion and strengthening of the deltoid muscle [7, 8]. The work force of the arm is allowed from the second month and sporting activities from 3 months.

Scapula fractures are very rare; they represents only 1% of all fractures [9]. In this group; these acromion fractures account only for 8-10% [10]. Mechanisms of injury of the acromion can be direct trauma; indirect trauma result of a dislocation of the humeral head; avulsion of the deltoid muscle. The most cases occur after a violent trauma and often in the public road accidents [11]. Fractures of the acromion good displaced or displaced can be consolidated with orthopedic treatment. Whereas treatment displaced fractures may be complicated by nonunion [12]. The indications for surgical treatment are still about discussion. By publication of Ogawa et al [13]. There are 4 Criteria of surgical treatment including symptomatic nonunion, concomitant ipsilateral scapula fracture, ≥ 1 cm of displacement upon radiographic assessment, and / or has multiple disruption of the superior shoulder suspensory complex (SSSC). The SSSC is the bony and soft tissue of the shoulder girdle ring that suspends the upper extremity from the thorax (the glenoid process, acromion, acromioclavicular ligament, clavicle, coracoclavicular ligaments, and the coracoid process) [14].

Several technics for fixation of fractures have been described acromion, including tension band wiring for more distal fractures, plate fixation for fractures that are more proximal or through the acromial base and spine, interfragment screw fixation, as the case of our patient, plate fixation supplemented with interfragment screws, and fixation with Kirschner wires. According to the DASH score functional outcome is very satisfying to 6 months after ablation materiel. No local complications were reported especially calcification or early or late infection.

## Conclusion

This is a very rare case that combined an open acromion fracture to a supraspinatus fracture. Care of this type of lesion should be emergency operated with a solid osteosynthesis and tendon suture for early and appropriate rehabilitation.
